# Measuring Activity-Rest Condition by Using Two Different Accelerometer Devices in Trained and Retired Horses

**DOI:** 10.3390/ani16162570

**Published:** 2026-08-18

**Authors:** Claudia Giannetto, Francesca Aragona, Francesco Fazio, Elisabetta Giudice, Alessandro Zumbo, Giuseppe Piccione

**Affiliations:** Department of Veterinary Science, University of Messina, Via Giovanni Palatucci, 98168 Messina, Italy; clgiannetto@unime.it (C.G.); ffazio@unime.it (F.F.); egiudice@unime.it (E.G.); azumbo@unime.it (A.Z.); gpiccione@unime.it (G.P.)

**Keywords:** locomotor activity, resting, horses, accelerometer

## Abstract

To advance technological applications in animal welfare assessment, this study evaluated resting activity as a component of total locomotor activity (TLA) in horses using two different accelerometer devices: a collar-mounted device containing a single-axis accelerometer that records physical movement, and a three axes accelerometer that classifies the measured physical activity into nine different activities. Ten clinically healthy Italian saddle horses were enrolled in the study and divided into two groups on the basis of their lifestyle. Group A consisted of regularly trained horses housed continuously in boxes, Group B comprised retired horses turned out in paddocks during the day and stabled in boxes at night. TLA was simultaneously monitored for 48 consecutive hours using both devices, in each horse. Two-way ANOVA applied to the one-axis and three-axis data showed a significant effect of group (*p* < 0.0001) and day in group A (*p* < 0.0001), with higher intensity values during day 1 compared to day 2 (*p* < 0.0001). A significantly greater amount of time spent in the “Lying/sitting rest” (recumbency) position was observed in group A (3.37 h ± 1.19 h) compared to group B (1.88 h ± 0.06 h), in both days of monitoring (*p* < 0.0001). A greater amount of time spent in the “Lying/sitting rest” (recumbency) position was observed during day 1 (4.16 h) compared to day 2 (2.58 h), in group A (*p* < 0.0001). Both devices detect the same qualitative pattern of group and day differences. Different amounts of activity were observed in trained and retired horses, with more rest periods in trained than in retired horses.

## Simple Summary

The assessment of locomotor and resting behavior is an important component of evaluating horse management. This study investigated total locomotor activity and resting time using a single-axis and a three-axis accelerometer device in horses. Ten clinically healthy Italian Saddle horses were divided into two groups: trained horses housed in boxes and undergoing weekly show-jumping sessions, and retired horses housed in paddocks during the day and boxes at night. Both devices simultaneously recorded activity for 48 consecutive hours. Significant differences were observed between groups and between monitoring days, with trained horses showing greater activity on the first day than on the second. Trained horses also spent significantly more time in recumbency position than retired horses, particularly during the first day of monitoring. Both devices detect the same qualitative pattern of group and day differences.

## 1. Introduction

A horse’s behavior provides useful information about its condition and is a good indicator of well-being. Horses have highly repetitive daily routines, so quantifying their behavioral activities over time can help assess their well-being. Among animals’ physiological behaviors, rest is one of the most important, as it plays a fundamental role in maintaining homeostasis and circadian systems restoration. Lying behavior is of particular interest in horses as changes in time budgets are understood to reflect a change to the animal’s environment or stress level. In horses, rest conditions include phases of physical and mental rest. During rest, the animal is in a motionless phase, characterized by both conscious and unconscious movements, whether standing or recumbent, during which it can enter the sleeping phase [1]. Rest activity is a component of the physiological behavior usually recorded within the total locomotor activity (TLA) assessment. In the context of TLA, the term “time-budget” refers to how an animal distributes its daily time among various activities. This distribution is often expressed as a percentage of the total time, reflecting the animal’s natural behavioral patterns and priorities [2]. In natural settings, horses allocate their time to grazing/foraging (67–75%), resting (including sleeping 15–25%), examining their surroundings (6–10%), or moving (8%), with the remainder dedicated to maintenance activities, social interactions, and reproduction [2]. In horses, rest exhibits polyphasic patterns, characterized by brief sleep intervals interspersed with periods of activity. In horses, the length of recumbence ranges from 11% to 20% of their time over 24 h, with lateral recumbence accounting for around 20% of the overall recumbence time [3,4]. The observation of locomotor activity and resting behavior is used to determine time budgets, which may give some indication for the assessment of horses’ welfare concerning management and housing. As an alternative to time-consuming direct observations, a common automatic measuring method is the use of accelerometers. Actigraphy is the use of an accelerometer to record bodily activity, particularly to assess the quantity and quality of sleep and irregular circadian rhythms [5].

In horses, the first study to find a method for long-term registration of horse behavior was conducted by Gill (1991) [6]. In later studies, one-axis actigraphy-based data loggers were used to analyze the total activity of horses. It has been demonstrated that in horses, TLA exhibits a 24 h fluctuation and can be influenced by external cues such as photoperiod, housing conditions, feeding schedules, or exercise [7,8,9,10]. Based on their locomotor activity patterns, horses can be classified as diurnal animals. In horses housed in stalls with or without paddocks, locomotor activity showed a diurnal pattern, and its acrophase always occurred in the middle of the photophase [11]. The daily rhythm of activity is influenced by the varying daylight of the seasons, without affecting the evidence of the acrophase, which remains constant over time and is independent of the time of sunrise [7]. Horses tend to be in recumbency position or sleep when they feel safe and comfortable in their environment [12]. Prolonged periods of rest can be an early sign of illness, so any changes in behavior should be carefully monitored.

Over the last two decades, the introduction of advanced technologies, such as wearable sensors and non-contact systems, has partially filled gaps in clinical practice. These tools, through continuous and non-invasive monitoring, allow for the collection of physiological and behavioral data, which can support detection of clinical issues. In particular, technological progress has driven a gradual transition toward automated methods for tracking domestic animal movement. A wide range of applications has emerged to enhance livestock management, evaluate working conditions, and safeguard animal welfare. Key innovations include precision grazing (PG) technologies, management software, and computer vision techniques for monitoring grazing patterns. Furthermore, modern systems utilize electronic identification (EID), accelerometer and GPS-based motion tracking, acoustic analysis, drones, and implantable sensors for real-time health and welfare assessments [13,14].

Three-axis accelerometers have been shown to be suitable for recording motor behavior in cows and in adult horses [15,16]. Triaxial accelerometers offer a non-invasive method for recording micro-scale bodily movements in real-world settings. Currently utilized in veterinary research, this technology lays the foundation for highly accurate, future monitoring of both livestock and companion animal activities [17]. The three axis accelerometer, using a predefined algorithm, combines the orientation of the sensor relative to gravity with the observed acceleration to identify different classifications of locomotor activity: lying down, sitting, standing, intense activity, moderate activity, immobility, and other activities. In horses, measuring lying behavior is an essential component of equine welfare assessment. The application of suitable devices can identify horses with low recumbency time, and decreased locomotion time budgets, indicating a general compromise of well-being [12].

The present study aimed to evaluate rest activity conditions as part of total locomotor activity in regularly trained and retired horses using two different devices, a single-axis accelerometer and a three-axis accelerometer.

## 2. Materials and Methods

### 2.1. Animals

Ten clinically healthy Italian saddle horses in good nutritional condition (the Henneke Body Condition Scoring System: 5) were enrolled from the same private training center. Before the study began, a clinical examination was conducted by a vet belonging to the research team to assess their health status based on respiratory rate, heart rate, body temperature, appetite, fecal consistency, and hematological and hematochemical parameters. The horses were divided into two equal groups on the basis of their lifestyle. Group A (n = 5), consisting of 3 non-pregnant females and 2 geldings (7–13 years old), regularly trained and housed in boxes. Group B (n = 5), including 2 non-pregnant females and 3 geldings (10–14 years old) retired from sport, housed in a paddock during daytime and in boxes during nighttime, not subjected to training. The animals of group A were trained until the day before the start of the study. All horses were trained for 1 h each day including walking, trotting, galloping and obstacle jumping with the same training protocol for show jumping. During the recording period, the horses of group A were not exercised.

TLA was simultaneously recorded for 48 consecutive hours using the two accelerometer devices, in all horses at the same time.

Thermo-hygrometric recordings were conducted during the entire study using a data logger (Gemini, Chichester, West Sussex, UK; Software Tinytag Explorer Vers. 4.6). Ambient temperature was within the seasonal range of the period (12–17 °C; Rh 63–66%), and no rain or wind was observed.

One single-axis accelerometer (Actiwatch-Mini^®^, Cambridge Neurotechnology Ltd., Fenstanton, Cambridgeshire, UK) used for recording physical activity was placed on a halter according to the previous literature [7,8,9,10,11]. It is an actigraphy-based data logger that records a digitally integrated measure of motor activity. This data acquisition system employs miniaturized accelerometer technologies; specifically, the Actiwatch utilizes a piezoelectric accelerometer configured to capture the integration of intensity, quantity, and duration of movement in all directions. The resultant voltage is converted and recorded as an activity count in the memory unit of the Actiwatch. The maximum sampling frequency is 32 hertz. It is important to stress that due to this improved way of recording activity data, there is no need for sensitivity settings as the Actiwatch unit records all movements over 0.05 g. Activity was monitored in 5 s intervals and quantified in arbitrary units. Actograms, a type of graph commonly used in circadian research to plot activity against time, were drawn using Actiwatch Activity Analysis 5.06 (Cambridge Neurotechnology Ltd., Fenstanton, Cambridgeshire, UK).

The three-axial accelerometer (Sens Motion^®^, Copenhagen, Denmark) was affixed to the right forelimb, at the lateral face of the shin region. The activity was evaluated by measuring the intensity of the executed motion and its position. Raw acceleration is calculated as the average magnitude of movement on a high-pass filtered 3-axis accelerometer measurement at 12 Hz sampling frequency. Data were sampled in 5 s intervals. The utilized hardware version was SENS motion plus version 1.3.0, accompanied by sensor firmware version 1.0.2. An algorithm integrates direction and acceleration to provide various activity classifications (Table 1). The motion category “Lying/sitting rest (recumbency)” was considered for rest activity estimation.

The study was approved by the Ethical Committee of University of Messina (Approval Code: 06/2023 of 21 March 2023).

### 2.2. Statistical Analysis

Actiwatch Activity Analysis 5.06 (Cambridge Neurotechnology Ltd., Fenstanton, Cambridgeshire, UK) was used to calculate the average amount of activity (bout of activity/hour) during the light and dark phases of data obtained from the one-axis accelerometer. SENS motion^®^ app and SENS motion^®^ online visualization tool was used to collect the recorded data from the three-axis device. Due to the large amount of data, the collected recordings obtained by both devices were grouped in 5 min intervals using Microsoft Excel spreadsheets. The data obtained from the two devices were not compared with each other as the two devices recorded at different frequencies.

Data exhibited a normal distribution (Kolmogorov–Smirnov test, *p* > 0.05) and were presented as mean ± standard deviation (n = 5). To investigate the effect of group and day of monitoring, a linear mixed-effects model (LMM) was applied to the raw data recorded every 5 min with the one-axis device (288 data points), the 5 s intervals data recorded with the three-axis device (17,280 data points), and the “lying/sitting rest (recumbency)” category. To account for the high-frequency dependence between repeated observations from the same individual, the individual horse (n = 5 per group) was included in the model as a random intercept.

The statistical analysis was performed using STATISTICA 8.0 software (StatSoft, Krakow, Poland). *p* < 0.05 was considered statistically significant. The investigators evaluating the actograms were blinded to the assigned group or other activity of each horse as they evaluated the data.

## 3. Results

**Figure 1:**
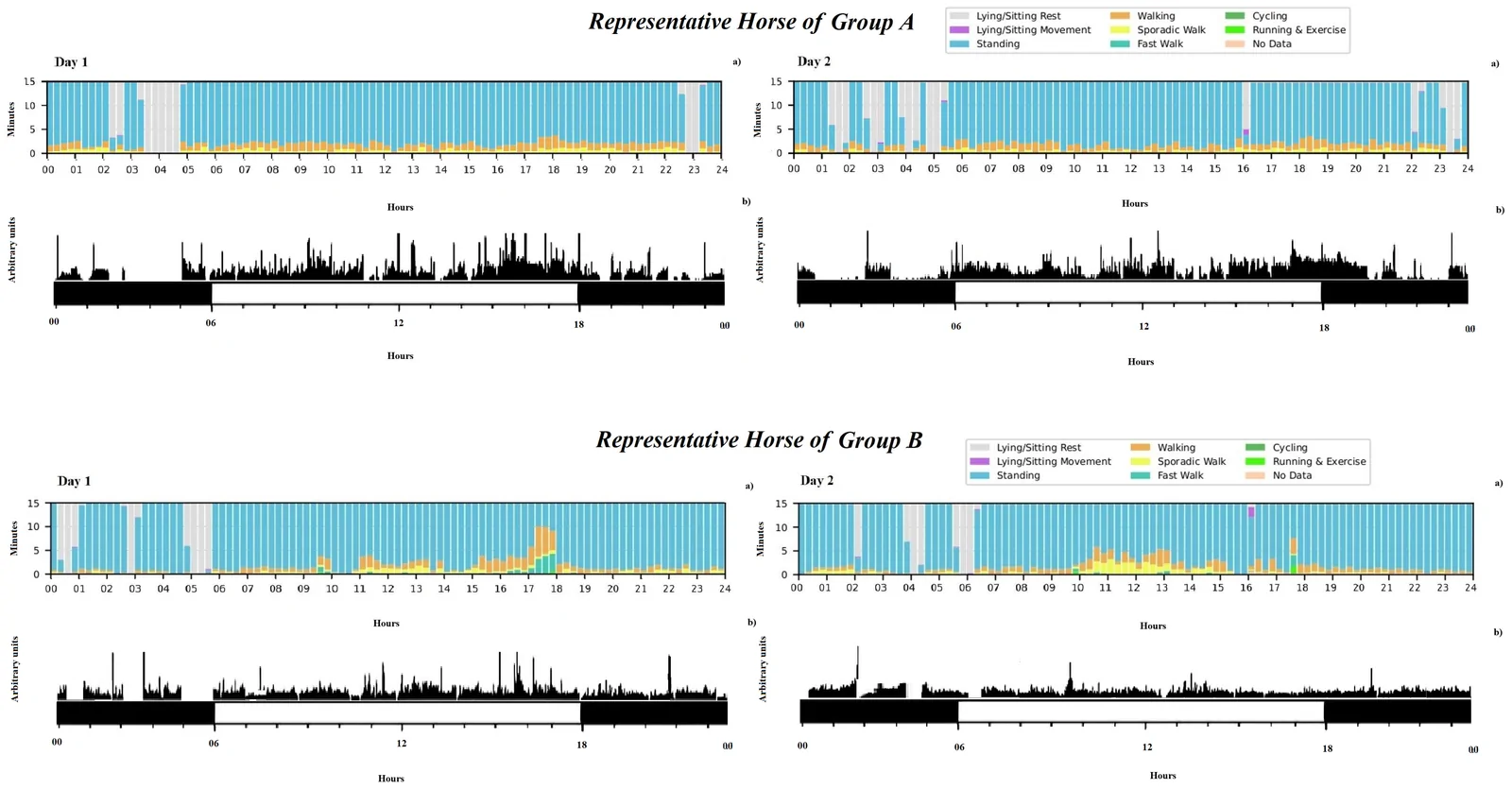
Total locomotor activity recorded with the one- and three-axis device of a representative horse of group A and B recorded during day 1 and day 2. (**a**) represents the three-axis device recording grouped in 5 min intervals including each activity category as shown in the legend. (**b**) represent the one-axis devise recording expressed in arbitrary unit; white and black bars indicate light and dark phases, respectively.

The TLA of a representative horse of groups A and B recorded with both devices is shown in Figure 1. Actiwatch Activity Analysis indicated in both groups a higher amount of activity during the light phase (Group A: 334.93 ± 26.07; Group B: 225.32 ± 13.45 arbitrary unit) than the dark phase (Group A: 149.79 ± 9.68; group B: 225.32 ± 16.12 arbitrary unit) of the daily photoperiod, on both days of monitoring.

The application of the LMM on the single-axis accelerometer data showed a significant effect of group (F_(1,287)_ = 146.82; *p* < 0.0001) and day of monitoring (F_(1,287)_ = 7.04; *p* < 0.01). The application of the two-way ANOVA on the three-axis accelerometer data showed a significant effect of group (F_(1,17276)_ = 194.79; *p* < 0.0001) and day (F_(1,17276)_ = 74.74; *p* < 0.0001). The application of the Bonferroni post hoc comparison test applied on the single-axis accelerometer data showed higher intensity values in group A than group B (*p* < 0.0001) on day 1 and lower intensity values in group A than group B on day 2 (*p* < 0.0001). In group A, significant higher intensity values were observed on day 1 compared to day 2 (*p* < 0.0001) as shown in Table 2. No difference between days was observed in group B (*p* = 0.99). The Bonferroni post hoc comparison test applied on the “Lying/sitting rest (recumbency)” category obtained by the three-axis accelerometer data showed a significantly greater amount of time spent in “Lying/sitting rest (recumbency)” position in group A compared to group B in both days of monitoring (*p* < 0.0001). A significantly greater amount of time spent in “Lying/sitting rest (recumbency)” position was also observed in group A on day 1 compared to day 2 (*p* < 0.0001), as shown in Table 2. No significant difference was observed between days in group B (*p* = 1).

## 4. Discussion

Rest plays an essential role in the restoration of the homeostatic and circadian systems [18]. Horses are obligate, non-ruminant herbivores that evolved as prey animals. Due to this evolutionary history, they possess a highly reactive, skittish nature designed to detect threats and escape, utilizing speed and herd behavior for survival. This evolutionary condition significantly influences the period and mode of rest in this species; this is also in relation to the habitat in which it is found. It has been reported that horses kept in a box, during the day, only engaged in a stand rest position [19]. This pattern may be due to the management of the stable, which affects the resting pattern of the horses. During the day, the horses were occupied with daily stable management, where they were let out into the paddock, groomed, and went for riding classes. Horses may also feel insecure during the day because of the presence of people around them, as they are prey animals [20]. The use of automated tools for recording the temporal trend of a specific parameter helps to establish good management practices and makes it possible to speed up the monitoring of these assessments and make them increasingly accurate and reliable. The activity count measured by the one-axis accelerometer device was widely investigated in horses; it provides generically information on all the daily behaviors, such as feeding, drinking, walking, grooming, and small movements during sleep/rest. A triaxial accelerometer is a non-invasive means of recording all small-scale body motions of an animal, providing a more detailed classification of activity categories. It can split each movement into different categories according to intensity and device position, identifying rest condition as the hours that the animal spent lying down in a state of immobility (lying/sitting rest).

Despite the limited number of subjects enrolled, and the 48 h duration for each photoperiod, from a visual analysis of the obtained actograms, a greater activity is observed during the daytime, compared to the night hours, as confirmed by the analysis of the obtained data. During the dark period, there are several activity peaks, mostly with lower intensity and shorter than during the light period, with several cycles of rest, in accordance with the previous studies conducted in horses [9,21]. Although sleep cycles of minimal intensity were observed, this type of recording does not provide accurate information on whether the horse is sleeping or resting in an upright or recumbent position [7,9,22]. Despite the two devices not being placed in the same position, but where indicated by the literature and suggested by manufacturer, the cycles of rest/activity observed in the actograms visually correspond to the nine categories graph obtained with the three-axis accelerometer. The analysis of data reported the same statistical differences in the amount of TLA recorded with the two different accelerometers, with a difference between the two groups. It is well known that in horses, there is a different amount of activity in animals housed in boxes compared with those housed in paddocks [23]. The comparison between two days of monitoring allows evaluation of the stability of the recorded activity, previous study reported the inter-subject and intra-subject variability of total locomotor activity in horses [23].

The analysis of the “Lying/sitting rest (recumbency)” category showed a rest amount in accordance with previous studies. Compared to other mammals, horses have a reduced rest duration, reaching a total amount of 3.82 h in 24 h [24]; they lie down only for short periods, and most of their rest occurs in an upright position. In particular, the present results showed significantly higher values of hours spent in the “Lying/sitting rest” position in group A horses compared to horses in group B, on both days of monitoring. A possible explanation of the significant difference found between the groups can be attributed to the fact that horses in group A had undergone routine physical exercise until the day before the start of the study, requiring more rest for recovery, compared to horses in group B which were retired animals no longer subject to training. Rumpel et al. (2021) [25] indicated that continuously trained horses engaged in more sedentary physical activity at night, resulting in increased rest compared to intermittently trained horses, who predominantly participated in light, moderate, and vigorous physical activities. Enhanced physical activity was also correlated with confinement [23]. Trained stabled horses exhibited altered nighttime behavior in contrast to retired horses. Consequently, exercise likely has several impacts on horses, both physically and mentally, resulting in increased physical tiredness [25].

Although, the simultaneous recording of trained and retired horses, housed in different conditions could be confusing in determining whether the observed differences in locomotor activity and resting behavior result from training status, housing management, or a combination of both.

Also, it is assumed that stabled horses have better housing and living conditions than other domesticated farm species, but box stall confinement results in limited and controlled exercise, that in saddle horse induce a reduction in motor activity [23].

The heightened energy consumption during training likely necessitates extended periods of recumbency in trained horses during the early morning hours. This can also be supported by the statistically significant difference in hours spent in the “Lying/sitting rest” position in horses from group A, which was higher on day 1 than on day 2, during which they did not engage in any physical activity. Sleep variability in horses may be due to differences in age, diet, daily exercise or their ability to adopt a recumbent position. The resting phase is characterized by NREM sleep, which in horses can be achieved both in a standing and lying position; and by REM sleep, which can only be achieved in a lying position [26]. Therefore, increasing the hours spent lying down in domesticated horses kept in stalls, compared to free-ranging horses, can positively impact their well-being and athletic performance by increasing the likely hours of REM sleep [1]. Whilst most sleep occurs at night; stabled horses do rest during the day.

## 5. Conclusions

Both the one axis accelerometer and the three-axis accelerometer showed a different amount of activity in trained and retired horses, with more rest periods in trained than in retired horses. ‘Differences in locomotor activity and resting time were observed between trained, continuously stabled horses and retired horses with daytime paddock access; however, the respective contributions of exercise and housing conditions cannot be separated in the present study. The use of three axis accelerometer improves the knowledge about the time budget in horses living in housing conditions. The classification in categories of locomotor activity allows to have an objective, continuous profile of daily horses’ behavioral patterns. The use of automated tools to record the horse’s behavior helps to establish good management practices. Knowledge about TLA and resting time is of great importance for the optimum management of equines. Further studies are necessary to investigate all categories and to find a reference range in various housing conditions.

## Figures and Tables

**Table 1:** Activities divided into 9 categories based on their intensity count calculated as the average magnitude of movement based on high pass filtered 3 axis accelerometer at 12 Hz sampling frequency done at 5 s interval. The intensity over a 5 s interval is normally between the values of 0 and 100, where 100 represents the highest intensity.

Activity Categories	Description of Categories
Resting (Lying/Sitting rest)—(recumbency)	The sensor is horizontal (±45 degrees), intensity < 2. Rising intensity the movement may be classified in lying (no movement) or sitting (recumbency) (no/little movement).
Lying or Sitting Movement	The sensor is horizontal (±45 degrees), intensity > 2. All movement of the subject in horizontal lying or sitting position (recumbency).
Sporadic Walking (upright sporadic walk)	The category includes epochs where an activity is starting towards the end or taking few steps and standing. The vector magnitude is above the movement threshold but below moderate intensity.
Walking (upright walk)	Intensity above 0.2 Hz but below the threshold for moderate activity. It includes asymmetrical movements.
Moderate Intensity	Activity above the walking intensity interval continuously for at least 3–9 s (slow running) lower than high intensity.
High Intensity/Running	Walking activity above the moderate intensity interval for a period of 3–9 s continuously.
Biking	Repetitive pattern recognized with a frequency above 0.2 Hz. The intensity must be above the measurement threshold. Both legs move continuously and movements are symmetrical.
Steps Taken Number of steps	The number of steps is counted during sporadic walking, walking or high intensity activities. The «Steps» category is further divided: Low (Step taken during low intensity walking where continuous frequency can be recognized in a 5 s interval). Medium (Step taken during sporadic walking with no continuous frequency during 5 s interval recognized as 2 steps per 5 s). High (Step taken during continuous walking activity and training with a frequency of motion during 5 s interval).

**Table 2:** Mean ± standard deviation (n = 5) of locomotor activity recorded with one-axis accelerometer device expressed in arbitrary unit and the Lying/sitting rest condition recorded with the three-axis accelerometer device expressed in seconds (s) and hours (h) in both groups during both days of recording and their statistical differences: * indicates differences between days (*p* < 0.0001) of the same parameter ^A^ indicates differences between group (*p* < 0.0001) of the same parameter.

**Activity Parameters**	**Day 1**	
	**Group A**	**Group B**
One-axis recording (Arbitrary unit)	252.1 ± 107.1 *^A^	182.1 ± 108.7 ^A^
Lying/sitting rest condition (s)	14,958.75 ± 2248 ^A^	7015 ± 1180 ^A^
Lying/sitting rest condition (h)	4.16 ± 0.37 *^A^	1.95 ± 0.20 ^A^
	**Day 2**	
	**Group A**	**Group B**
Locomotor activity (Arbitrary unit)	134 ± 110.6 *^A^	203.4 ± 101.7 ^A^
Lying/sitting rest condition (s)	9291.25 ± 1798 ^A^	6566.67 ± 1101 ^A^
Lying/sitting rest condition (h)	2.58 ± 0.30 *^A^	1.82 ± 0.18 ^A^

## Data Availability

The datasets generated during and/or analyzed during the current study are available from the corresponding author on reasonable request.
